# Clinicopathologic and prognostic significance of tumor-associated macrophages in cervical cancer: a systematic review and meta-analysis

**DOI:** 10.1007/s12094-024-03587-1

**Published:** 2024-07-08

**Authors:** Xinmei Lin, Jijie Zhan, Ziting Guan, Jingwei Zhang, Tian Li, Li Zhong, Changlin Zhang, Miao Li

**Affiliations:** 1 Department of Gynecology, The Seventh Affiliated Hospital of Sun Yat-Sen University, Shenzhen, China; 2 Guangdong Provincial Key Laboratory of Digestive Cancer Research, Department of Scientific Research Center, The Seventh Affiliated Hospital of Sun Yat-Sen University, Shenzhen, China

**Keywords:** Cervical cancer, Tumor-associated macrophages, Meta-analysis, Prognosis

## Abstract

**Objectives:**

The role of tumor-associated macrophages (TAMs) in cervical cancer (CC) remains controversial. Here, we report a meta-analysis of the association between TAMs infiltration and clinical outcomes.

**Methods:**

PubMed, Embase, Web of Science, and CNKI were searched systematically from inception until December 20, 2023. Studies involving TAMs and prognosis, clinical, or pathological features were included. Quality assessments of the selected studies were assessed. The fixed-effect or random-effects model, standard mean difference (SMD), odds ratios (OR), or hazard ratios (HR) with 95% confidence intervals (CIs) were used as the effect size estimate.

**Results:**

26 eligible studies with 2,295 patients were identified. Our meta-analysis revealed that TAMs were overexpressed in CC (OR = 12.93, 95% CI = 7.73–21.61 and SMD = 1.58, 95% CI = 0.95–2.21) and that elevated TAM levels were strongly associated with lymph node metastasis (LNM) (SMD = 0.51, 95% CI = 0.90–2.01) and FIGO stages (SMD = 0.46, 95% CI = 0.08–0.85). Subgroup analysis indicated a significant positive correlation between LNM and TAMs density in tumor stroma, but not in cancer nests (SMD = 0.58, 95% CI = 0.31–0.58). Furthermore, in early stage, a stronger correlation exists between LNM and TAM density (SMD = 1.21, 95% CI = 0.75–1.66). In addition, it revealed that patients with high TAMs expression had poorer overall survival (OS) (HR = 2.55 95% CI = 1.59–4.07) and recurrence-free survival (RFS) (HR = 2.17, 95% CI = 1.40–3.35).

**Conclusions:**

Our analyses suggest that a high density of TAMs predicts adverse outcomes in CC.

**Supplementary Information:**

The online version contains supplementary material available at 10.1007/s12094-024-03587-1.

## Introduction

Cervical cancer (CC) is one of the most common gynecological tumors [1]. In 2020, there were 109,700 new cases and 59,000 deaths of CC in China [2]. Although vaccinations, radical surgery, and radio-chemotherapy have been widely used to prevent and treat CC, patients with advanced-stage, recurrence, and metastasis often lack effective treatment options, leading to poor overall prognosis. In recent years, with immunotherapy promoted in clinical practice, a new era in the treatment of CC has emerged [3].

Tumor-associated macrophages (TAMs) are an important part of the tumor microenvironment (TME) [4]. TAMs are double-edged swords with dual roles in cancer. TAMs are crucial for tumor immune surveillance in the early stages of tumor development. Nonetheless, accumulating data indicates that TAMs complicate cancer treatment by promoting tumor growth in advanced stages [5]. TAMs play a role in immunosuppression, metastasis, angiogenesis, extracellular matrix remodeling, cancer cell proliferation, and resistance to chemotherapeutic medications and checkpoint blockade immunotherapy [6]. Therefore, a clear understanding of TAMs is essential for effective cancer treatment.

Findings relating to the role of TAMs in CC remain controversial [7]. For example, Heller et al. discovered that macrophage infiltration was strongly negatively associated with tumor stage, but not with tumor grade or histological lymph node status [8]. In contrast, it has been reported that high CD163 + macrophages were significantly associated with higher FIGO stage and lymph node metastasis (LNM) [9]. Furthermore, Kawachi et al. discovered that CD204 + TAMs were significantly associated with shorter disease-free survival [10], whereas Davidson et al. demonstrated that macrophage density was not associated with survival in cervical cancer patients [11]. Current studies have shown that the characteristic molecular markers of TAMs include CD68, CD163, CD204, and CD206 et al. [12, 13]. However, few biomarkers have been successfully translated into clinical practice. Therefore, we conducted a study to validate the association between TAMs and clinicopathological parameters as well as prognosis in CC, and then performed a meta-analysis to evaluate the role of different types of TAMs in the TME of CC by pooling data from 26 eligible studies.

## Materials and methods

To clarify the association between TAMs infiltration and clinical outcomes, we followed the PRISMA Statement guidelines to design, analyze, and report our meta-analytic findings [14]. Only the study-level summary data were used for the analyses.

### Search strategy

We comprehensively and systematically searched the PubMed, Embase, Web of science, and Chinese National Knowledge Infrastructure (CNKI) databases to obtain a preliminary list of relevant studies. The literature search was carried out from inception to December 20, 2023. We identified studies using Medical Subject Heading (MeSH) terms and corresponding keywords, including “Uterine Cervical Neoplasms”, “Tumor-Associated Macrophages”, etc. (Supplementary Table S1). There were no restrictions on language and study design. We also manually checked the bibliographies of previous reviews and references in all selected studies. All the references were exported and managed using Zotero 6.0.30 software.

### Inclusion and exclusion criteria

The inclusion criteria were: (1) studied patients with CC were pathological examination confirmed; (2) TAMs were measured by immunohistochemistry (IHC), and the markers included CD68, CD163, CD204, CD206; (3) correlation of TAMs with prognosis, clinical, or pathological features was reported; (4) the studies were prospective or retrospective cohort studies or case–control studies; and (5) more than 6 points of Newcastle–Ottawa Scale (NOS) score were considered eligible for inclusion.

The exclusion criteria were: (1) reporting of duplicate or overlapping data; (2) animal experiments, case reports, reviews, meta-analysis, conference abstracts; (3) sample size less than 30; (4) without sufficient data for estimating the correlation; and (5) full text is not available.

### Data extraction

Two authors (Xinmei Lin and Ziting Guan) independently selected and extracted the required data from all eligible studies. The following information was extracted: the first author’s name, country, publication year, study period, sample size, age, biomarkers, distribution, and density of TAMs, LNM, FIGO stage, etc. We also collected the prognostic information, including overall survival (OS) and recurrence-free survival (RFS). The hazard ratios (HRs) and 95% confidence interval (CI) were extracted from Kaplan–Meier (KM) plot using GetData Graph Digitizer (free software downloaded from: https://getdata.sourceforge.net/download.html) and estimated from KM curves using the method described by Parmar et al. [15]. The corresponding author of study was contacted to request any unclear or missing data. All disagreements were settled by a face-to-face consultation with a third author.

### Quality assessment

The Newcastle–Ottawa Scale (NOS) was used to assess the quality of each individual study; this was performed independently by two authors (Xinmei Lin and Jingwei Zhang). The NOS comprises three quality parameters: selection (0–4 points), comparability (0–2 points), and outcome assessment (0–3 points).

### Statistical analysis

To assess the heterogeneity of the included trials, Cochran’s Q test and Higgins-squared statistic were undertaken. If statistical heterogeneity was significant (*P* < 0.10 or I^2^ > 50%), the random-effect model was used. Otherwise, a fixed-effects model was used in meta-analysis. To investigate possible sources of between-study heterogeneity, we performed meta-regression and subgroup analyses based on biomarkers or distribution of TAMs. Additionally, to explore the heterogeneity and evaluate the stability of the results, we performed sensitivity analysis by excluding each individual publication and altering the statistical method. In cases where there was moderate heterogeneity among the meta-analysis results (I^2^ close to 50%, *P* slightly greater than 0.05), random-effects models were employed to assess the stability of pooled results obtained from fixed-effects models. Additionally, when the number of studies available was > 10, potential publication bias was assessed by the Egger’s test [16].

The odds ratio (OR) was used as the effective quantity in the counting data and the standard mean difference (SMD) was used as it in the measurement data. The SMD cut-off values of 0.2, 0.5, and 0.8 were used for small, medium and large effect sizes. While the HRs with 95% CI were used to evaluate the correlation between the TAMs density and survival. A two-tailed *P*-value < 0.05 was considered statistically significant. All analyses were carried out using the software Review Manager 5.4 and Stata 12.0.

## Result

### Selection and characteristics of studies

**Fig. 1:**
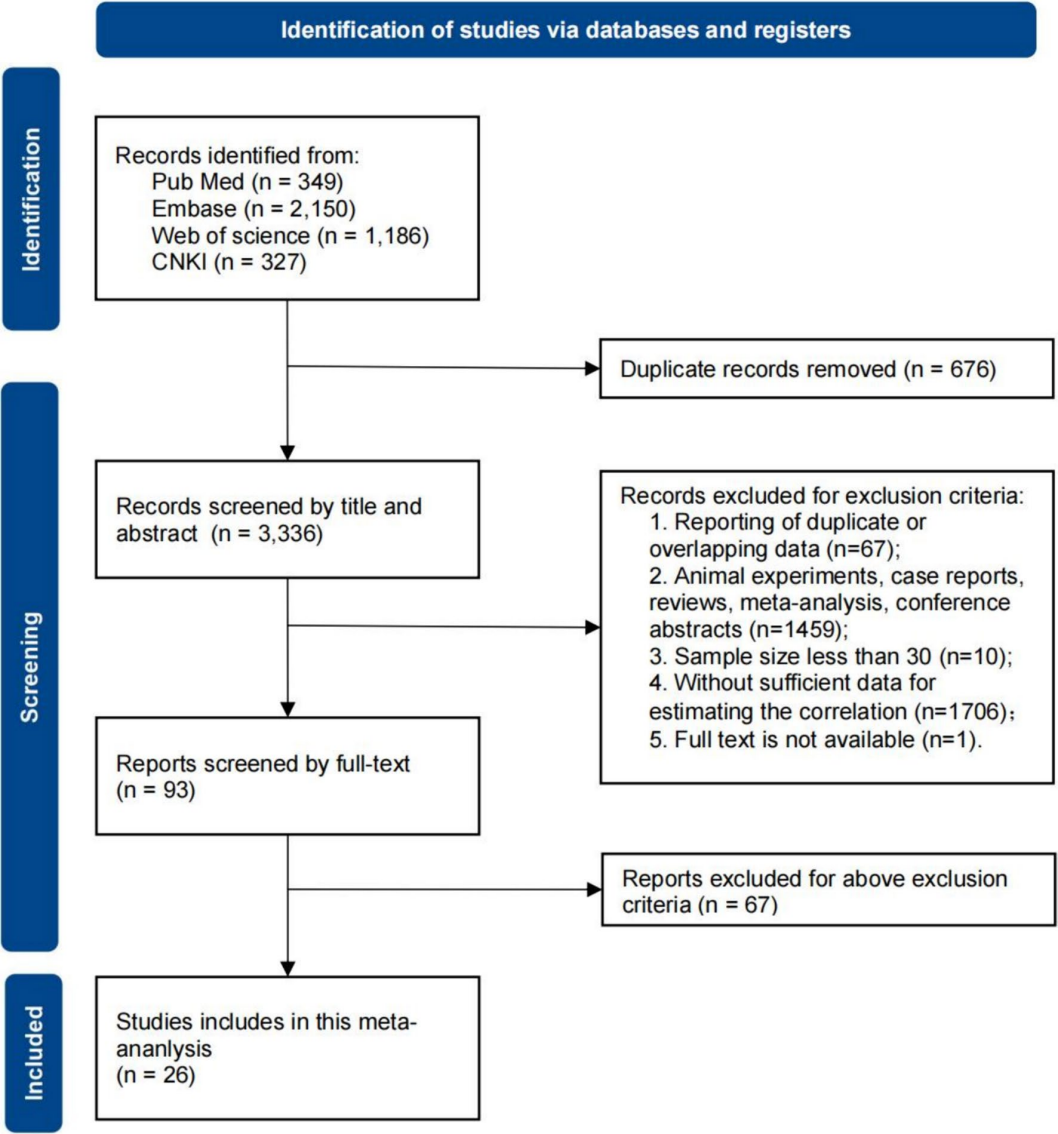
Flow diagram of the meta-analysis process

Through the preliminary screening of PubMed, Embase, Web of Science, and CNKI searches, 3336 studies were identified after we eliminated 676 duplicate documents. 3243 of them were discarded after examining the qualifying requirements based on the mentioned inclusion and exclusion criteria. 67 articles were further excluded after reading the whole texts and a total of 26 articles were finally included in the analysis [9–11, 17–39]. The PRISMA flow diagram shows the complete review process from the original search to the final selection (Fig. 1).

Included articles were published between 1999 and 2023 in four countries (China, Japan, Denmark, Israel), enrolling a total of 2295 patients with diagnoses of cervical squamous cell carcinoma, cervical adenocarcinoma, and adenosquamous carcinoma of cervix. The age of the patients ranged from 22 to 87 years. All studies were retrospective studies. The TAM markers were detected by IHC. Twenty-three articles provided information on the clinical pathology characteristics including age, FIGO stage, tumor size, LNM, organization type, and HPV infection status. Five articles included prognostic information (OS or RFS) [20, 21, 25, 34, 38], with a median follow-up of 6.45 years. The main characteristics of the included studies are summarized in Table 1. Additionally, the study quality scores, assessed using the NOS, ranged from 6 to 8, with a mean of 7.07 (Table 1). All articles included in this study were of high quality. The detailed results of this quality assessment were shown in Supplementary Table S1.

**Table 1:** Characteristics of included studies

Study ID	First author	Country	Publication year	Study period	Sample size	Age median (range)	Histologic type	Methods	Markers	TAM distribution	Outcome assessment	NOS
Wang 2023	Wang, Yin	China	2023	NR	37	51(33–67)	NR	IHC	CD68	Invasive margin, TC, TS	OS	7
Guo 2021 (1)	Guo, Fan	China	2021	2017–2019	100	NR	NR	IHC	CD68; CD163	TS	Age, LNM, HPV, Tumor size, FIGO	7
Liu 2021	Liu, Juan	China	2021	2015–2016	45	52(28–80)	SCC; AC; ASC	IHC	CD206	TS	Tumor size, LNM, FIGO	7
Zou 2021	Zou, Ruanmin	China	2021	2015–2018	406	NR	SCC; NSCC	IHC	CD68;CD163	TC	LNM,OS,RFS	7
Cao 2020	Cao, L	China	2020	2009–2013	122	50(26–76)	SCC	IHC	CD68; CD163	TS	LNM	7
Ding 2020	Ding, Yan	China	2020	2018	58	45.53(25–65)	SCC; AC	IHC	CD68	Peritumoral stroma, TC	CC/N	8
Guo 2020	Guo, F	China	2020	2012–2014	120	NR	SCC	IHC	CD68; CD163	TS	Age, LNM, HPV, Tumor size, OS, RFS	7
Liu 2020	Liu, Min	China	2020	2016–2019	42	NR	NR	IHC	CD68	NR	CC/N,LNM	7
Ohno 2020	Ohno, A	Japan	2020	2008–2013	55	NR	SCC	IHC	CD206	Intratumoral	Tumor size	7
Chen 2019 (1)	Chen, R	China	2019	2014–2016	96	48(24–71)	SCC; AC; ASC	IHC	CD68; CD163	TC, invasive margin	LNM	7
Chen 2019 (2)	Chen, Xiaojing	China	2019	2011–2013	38	NR	SCC	IHC	CD163	TS	Age, LNM	7
Kawachi 2018	Kawachi, A	Japan	2018	2001–2014	148	NR	AS	IHC	CD204; CD68	TS	HPV	6
Liu 2018	Liu, Miaomiao	China	2018	2013–2016	65	50(35–82)	SCC	IHC	CD68	NR	CC/N, LNM	7
Yan 2018	Yan, Reming	China	2018	2011–2013	41	NR	SCC	IHC	CD163	Peritumoral stroma	CC/N, Age, LNM, FIGO	7
Zhou 2018	Zhou, Chao	China	2018	2011–2016	89	45(32–87)	NR	IHC	CD68; CD163	NR	LNM, OS	7
Chen 2017	Chen, X.-J	China	2017	2011–2013	130	NR	SCC	IHC	CD68; CD163	Epithelium, stroma	CC/N, Age, LNM, FIGO	7
Li 2017 (1)	Li, Y	China	2017	2016	109	NR	SCC	IHC	CD163	TS, peritumoral stroma	CC/N	7
Li 2017 (2)	li, Xuelian	China	2017	2012–2016	50	NR	SCC	IHC	CD68	NR	CC/N, LNM, umor size, FIGO	7
Shen 2017	Shen, Yifan	China	2016	2013–2015	60	NR	SCC; NSCC	IHC	CD68; CD163	TS	CC/N, Age, LNM	7
Carus 2014	Carus A	Denmark	2014	1990–2000	101	44(22–70)	SCC	IHC	CD163	NR	RFS	8
Ding 2014 (1)	Ding, Hui	China	2014	2005–2007	55	NR	SCC	IHC	CD68	TC, TS	Age, LNM, FIGO	7
Ding 2014 (2)	Ding, Hui	China	2014	NR	61	NR	SCC; AC	IHC	CD68	TC, TS	CC/N, Age, LNM, FIGO	7
Li 2014	Li, Yan	China	2014	2007–2013	93	44(25–68)	SCC	IHC	CD68	NR	CC/N, LNM	7
Zheng 2013	Zheng, Jianqiong	China	2013	2010–2012	40	NR	SCC	IHC	CD68	NR	CC/N, LNM	7
Liu 2005	Liu, Dongju	China	2005	1996–1998	59	39(25–68)	SCC; AC	IHC	CD68	TS	LNM, FIGO	7
Davidson 1999	Davidson, B	Israel	1999	1984–1996	75	47.8(27–83)	SCC	IHC	CD68	NR	FIGO	6

*SCC* cervical squamous cell carcinoma, *AC* cervical adenocarcinoma, *ASC* adenosquamous carcinoma of cervix, *NSCC* non-squamous carcinoma of the cervix, *NR* not reported, *IHC* Immunohistochemistry, *OS* Overall survival, *LNM* Lymph node metastasis, *CC/N* Cervical cancer and normal tissue controls, *NOS* Newcastle–Ottawa Scale checklist

### TAMs exhibited high expression levels in cervical *cancer*

**Fig. 2:**
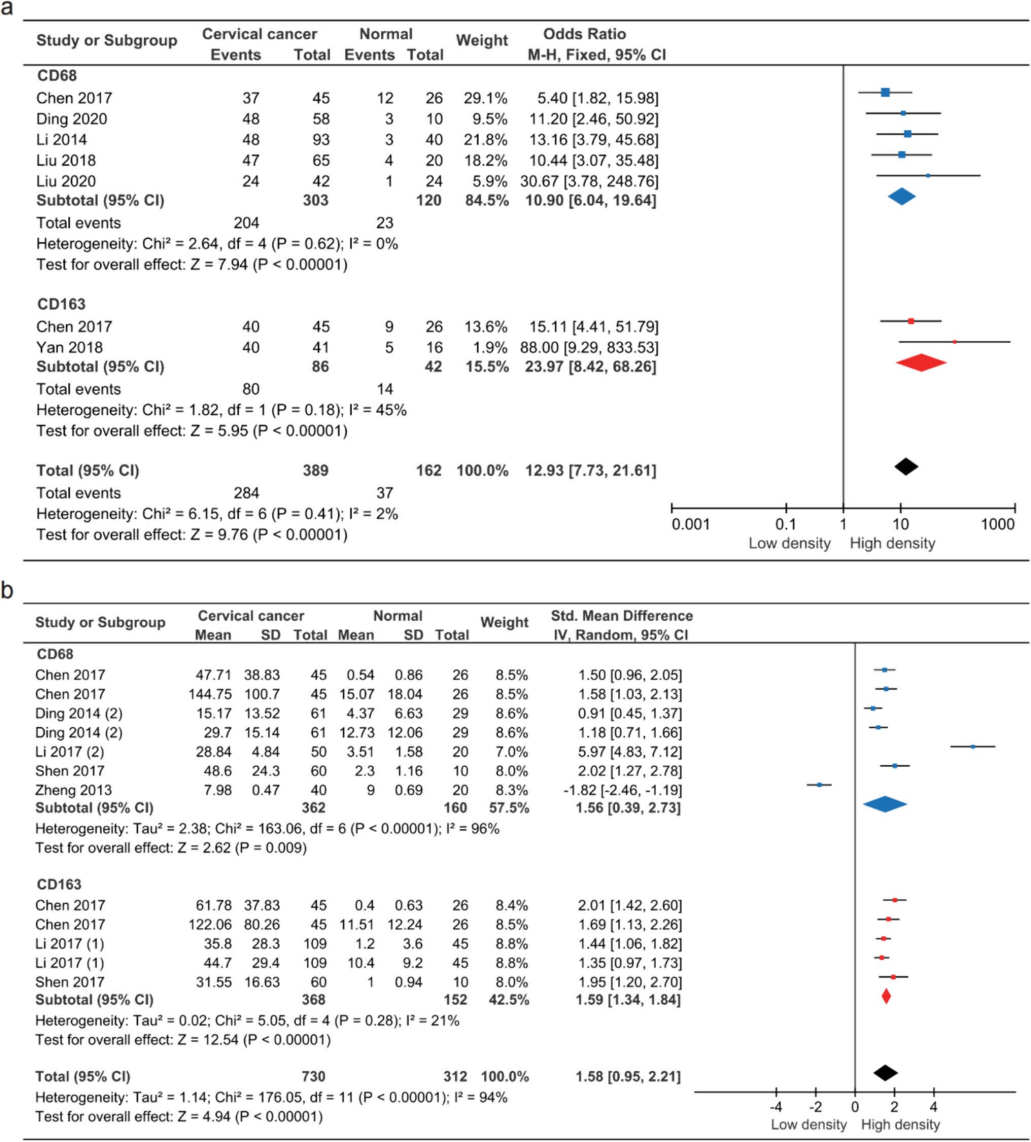
Forest plot of TAMs in CC and normal tissue. **a** TAMs expression in CC. **b** TAMs density in CC

Seven studies evaluated the expression of TAMs in CC and normal tissue. There was no significant heterogeneity between the two groups in terms of TAM expression (*P* = 0.41, I^2^ = 2%), necessitating the use of the fixed-effect model. A total of 12 studies investigated the density of TAMs in CC and normal tissue. Significant heterogeneity was found (*P* < 0.00001, I^2^ = 94%), prompting the use of a meta-analysis with a random-effect model. The results were significant (OR = 12.93, 95% CI = 7.73–21.61, *P* < 0.00001 and SMD = 1.58, 95% CI = 0.95–2.21, *P* < 0.00001), indicating a significant difference in the correlation between the expression and density of TAMs in CC and normal tissue. Results of subgroup analysis were consistent (Fig. 2).

### TAMs density and clinicopathological features

#### TAMs exhibited high expression levels in LNM cervical *cancer*

**Fig. 3:**
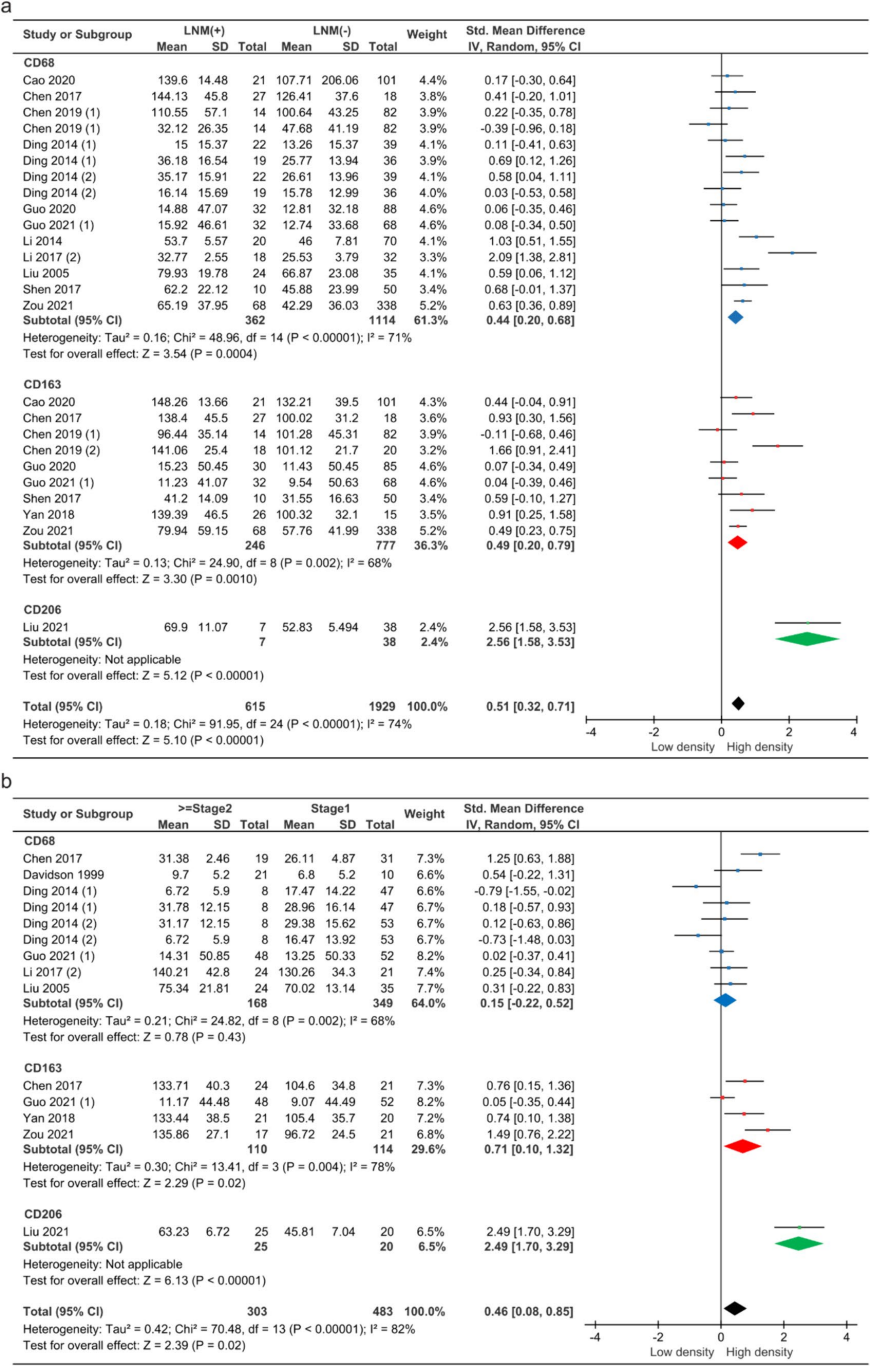
Forest plot of TAMs density and features. **a** TAMs density and LNM. **b** TAMs density and FIGO stage

The density of TAMs and LNM in CC was reported in 25 studies. Due to significant heterogeneity across studies (*P* < 0.00001, I^2^ = 74%), a random-effect model was used. The meta-analysis revealed a correlation between high TAM density and LNM, with a pooled SMD of 0.51 (95% CI = 0.90–2.01, *P* < 0.00001). Subgroup analyses showed a statistically significant correlation between CD68 + , CD163 + , and CD206 + TAMs and LNM in CC (Fig. 3 a).

Meta-regression analysis identified the distribution of TAMs and FIGO stage as potential sources of heterogeneity (*P* = 0.001 and *P* < 0.0001), suggesting that further subgroup analysis could be performed (Supplementary Fig. S1 a, b). A total of 20 studies analyzed the density of TAMs in tumor stroma (TS) or tumor center (TC). The study found a statistically significant correlation (SMD = 0.58, 95% CI = 0.31–0.58, *P* < 0.0001) between TAM density in TS and LNM. However, there was no statistically significant in the TC (Supplementary Fig. S1c). A total of 23 studies analyzed the density of TAMs in different FIGO stage. The results showed that there was statistical significance (SMD = 1.21, 95% CI = 0.75–1.66, *P* < 0.00001 and SMD = 0.24, 95% CI = 0.08–0.39, *P* = 0.003) in the correlation between the density of TAMs in stages 1–2 or stages 1–4 CC and LNM. Interestingly, the difference in TAMs density was greater in CC tissues at stages 1–2 compared to stages 1–4 (Supplementary Fig. S1d).

#### TAMs exhibited high expression levels in advanced cervical *cancer*

14 studies reported data on the density of TAMs and FIGO stage in CC. Due to significant heterogeneity among studies (*P* < 0.00001, I^2^ = 82%), the random-effect model was used. The meta-analysis revealed a trend of a correlation between high TAM density and FIGO stage, with a pooled SMD of 0.46 (95% CI = 0.08–0.85, *P* = 0.02). Subgroup analyses revealed a significant correlation between the density of CD163 + and CD206 + TAMs and the FIGO stage in CC (SMD = 0.71, 95% CI = 0.10–1.32, *P* = 0.02 and SMD = 2.49, 95% CI = 1.70–3.29,* P* < 0.00001) (Fig. 3b).

#### No association between TAMs density and age, tumor size, and HPV

Some studies reported the association between TAMs density and clinicopathological characteristics, including age, tumor size, and HPV infection status, but found no statistical significance (Supplementary Fig. S2).

### TAMs exhibited high expression levels in cervical *cancer* with poor prognosis

**Fig. 4:**
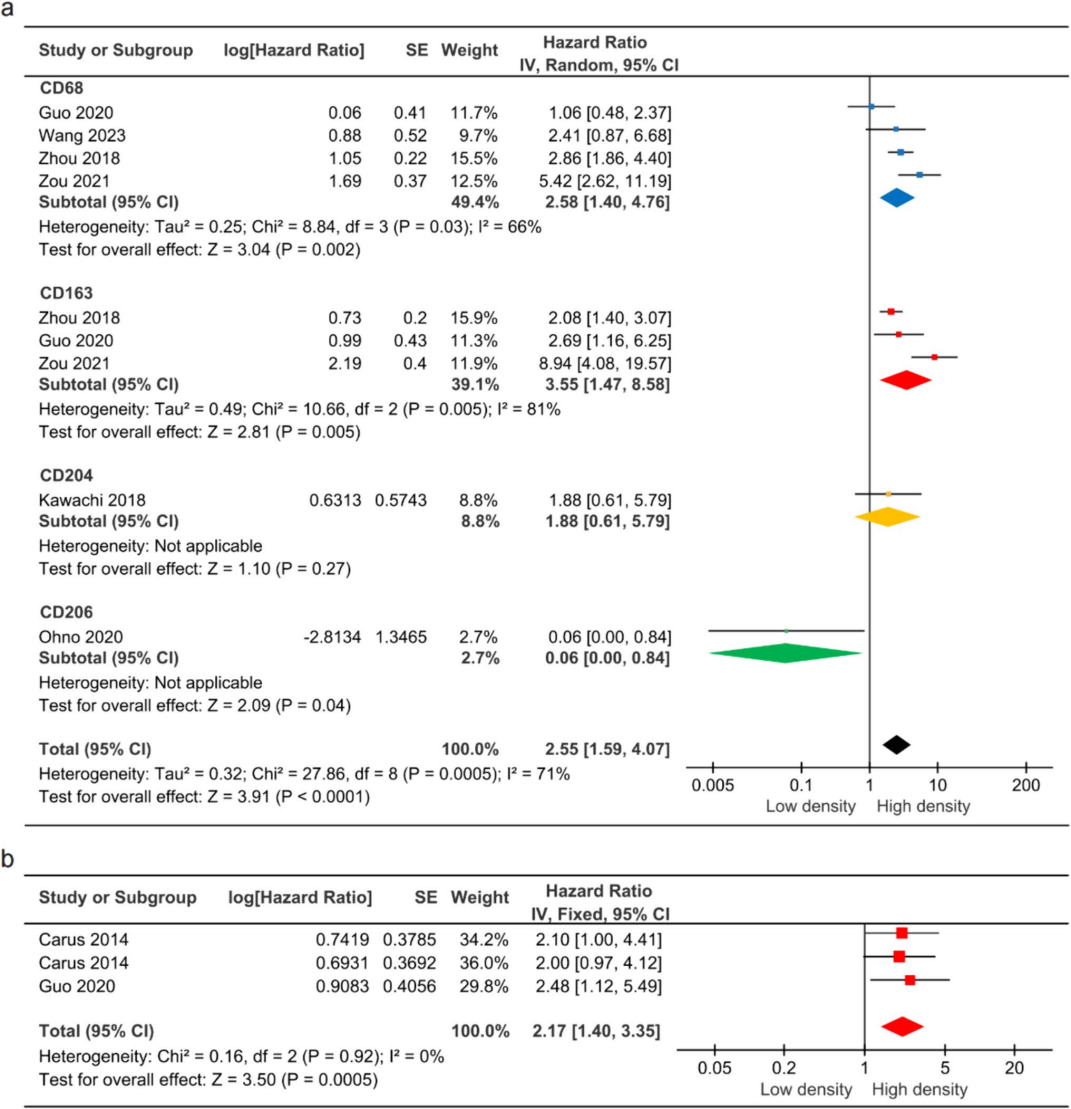
Forest plot of TAMs expression and prognosis. **a** TAMs density and OS. **b** TAMs density and RFS

Meta-analysis of 9 studies showed poorer OS in the high TAMs density group compared to the low TAMs density group, with a pooled HR of 2.55 (95% CI = 1.59–4.07, *P* < 0.0001) (Fig. 4a). Subgroup analysis revealed that the density of CD68 + , CD163 + , and CD204 + TAMs was significantly associated with poor OS, but the opposite was observed for CD206 + TAMs. Furthermore, three studies found a link between the density of CD163 + TAMs and RFS, showing statistical significance (HR = 2.17, 95% CI = 1.40–3.35, *P* = 0.0005) (Fig. 4b).

### Sensitivity analysis and publication *bias* test

Sensitivity analysis suggested that the results of each individual study on the pooled OR, SMDs and HRs were stable (Supplementary Table S3 and Supplementary Fig. S4). An official analysis utilizing Egger’s tests revealed that there was no publication bias in the meta-analyses pertaining to the relationships between TAM density and CC clinicopathological features (Supplementary Table S3 and Supplementary Fig. S4). While publication bias test was not performed for the meta-analyses of CC prognostic and other clinicopathological features, due to the insufficient number of studies available for a valid statistical test.

## Discussion

Our meta-analysis revealed that TAMs were overexpressed in CC and that elevated TAMs were strongly positively correlated with LNM and FIGO stage. However, there was no association between age, HPV infection, and tumor size. Subgroup analysis revealed a stronger correlation of LNM from CC with TAMs density in TS than that in TC, especially in early CC. In addition, the results showed that CC patients with high expression of TAMs had a poorer OS and RFS.

The association between TAMs and CC has gained a lot of attention recently; however, the precise role of TAMs remains elusive. This is the first meta-analysis to investigate the prognosis and characteristics in patients with CC exhibiting a high density of TAMs. Regarding the correlation between TAMs and LNM, our findings align with the earlier meta-analysis conducted by Guo et al. [24]. Furthermore, the study conducted by Troiano, Yuan, and Guo et al. revealed that a high-density infiltration of CD68 + or CD163 + TAMs was indicative of an unfavorable prognosis in head and neck squamous cell carcinoma, ovarian cancer, lung cancer and adult classical Hodgkin lymphoma [40–43]. Our meta-analysis results further validate that an increased density of TAMs in TME of CC is associated with a poorer prognosis.

Studies have shown that tumors actively recruit macrophages in the TME through various mechanism. For example, CCL8 derived from cancer cells binds to its receptor, C–C chemokine receptor 2 (CCR2) on macrophages, attracting macrophages [44]. TAMs exhibit diverse pro-tumor functions in the different stages of tumor development. TAMs release nitric oxide (NO) and reactive oxygen intermediates (ROI), resulting in DNA damage and genetic instability at the initiation stage [6]. In the later stage, TAMs down-regulate various functional molecules related to antigen presentation, such as major histocompatibility complex (MHC), costimulatory molecules and inflammatory factors [45]. Moreover, TAMs can inhibit the anti-tumor function of immune system by up-regulating the expression of immunosuppressive cytokines such as IL-10 and TGF-β, while down-regulating the expression of immune activating cytokines such as IL-12 [46, 47]. In addition, TAMs stimulate the division of preexisting lymphatic endothelial cells and vascular endothelial cells by increasing the production of vascular endothelial growth factor (VEGF), thereby inducing lymph angiogenesis and neovascularization, which supply nutrients to tumor tissue and promote tumor metastasis [48–50]. Interestingly, TAMs can also alter the immune cell composition in the TME. TAMs recruit Treg cells by secreting chemokine CCL22 [51], and PD-L1 + TAMs exert immunosuppressive effects by binding to PD-1 on T lymphocytes [52]. Our results are consistent with those studies of the mechanism of TAMs in the TME.

The meta-analysis of this study possesses strengths and implications, as well as certain limitations. First, the sensitivity analysis consistently demonstrated reliable and stable findings. However, despite employing sensitivity analyses and meta-regressions, there was significant heterogeneity in specific measurements that could not be fully elucidated. Although the mean study quality score according to NOS assessment was 7.07, all included studies were retrospective without any prospective ones. Additionally, one study had a follow-up period of less than 5 years and lacked direct HR data with a 95% CI, which could only be obtained from KM plot. Egger’s test did not identify publication bias in this meta-analysis; however, due to limited test power, the presence of publication bias in analyses comprising fewer than 10 included studies remains unknown. Moreover, different thresholds for TAM expression were utilized among the included studies, raising potential biases. Furthermore, this study primarily focused on IHC where technical biases may arise from reagents used, methods employed for evaluating positive results, operator skills, and other factors.

## Conclusion

In conclusion, this study demonstrates a clear difference in TAM infiltration between CC tumor tissue and normal cervical tissue. A high density of TAMs in the CC tumor microenvironment is associated with LNM and poor survival, providing valuable insights for predicting LNM and prognosis. Prospective clinical trials should evaluate TAMs to enhance the diagnosis and management of CC patients. Additionally, although the relationship between CC and TAMs has been partially uncovered, there are still significant gaps in our understanding. Further research is needed to investigate TAM processes, pathways, alongside the development of targeted therapies, which is essential for improving clinical outcomes for CC patients.

## Supplementary Information

Below is the link to the electronic supplementary material.

Supplementary file1 (DOCX 2530 KB)

## Data Availability

All the data in our systematic review were derived from published studies and, thus, are available. On reasonable request, they can be provided by the first author.
