# Supplementary material for: Clinicopathologic and prognostic significance of tumor-associated macrophages in cervical cancer: a systematic review and meta-analysis
Source: Clin Transl Oncol. 2024 Jul 8;27(1):351–62. doi: 10.1007/s12094-024-03587-1 (PMC11735494; doi:10.1007/s12094-024-03587-1)
Supplement: Supplementary file 1 — Supplementary file1 (DOCX 2530 KB) [file 12094_2024_3587_MOESM1_ESM.docx]

**Supplementary Table S1** Medical Subject Heading (MeSH) terms and corresponding keywords

|  | **#1** | **#2** |
| --- | --- | --- |
| **MeSH** | Uterine Cervical Neoplasms | Tumor-Associated Macrophages |
| **Keywords** | Cervical Neoplasm, Uterine | Macrophage, Tumor-Associated |
|  | Neoplasm, Uterine Cervical | Tumor Associated Macrophages |
|  | Uterine Cervical Neoplasm | Tumor-Associated Macrophage |
|  | Neoplasms, Cervical | tumour infiltrating macrophage |
|  | Cervical Neoplasms | cancer associated macrophage |
|  | Cervical Neoplasm | intratumoral macrophage |
|  | Neoplasms, Cervix | intratumoural macrophage |
|  | Cervix Neoplasm | TAM |
|  | Neoplasm, Cervix | TAMs |
|  | Cervix Neoplasms | tumor associated macrophage |
|  | Cancer of the Uterine Cervix | tumor infiltrating macrophage |
|  | Cancer of the Cervix | tumour associated macrophage |
|  | Cervical Cancer |  |
|  | Cancer, Cervical |  |
|  | Cervical Cancers |  |
|  | Uterine Cervical Cancer |  |
|  | Cancer, Uterine Cervical |  |
|  | Cervical Cancer, Uterine |  |
|  | Uterine Cervical Cancers |  |
|  | Cancer of Cervix |  |
|  | Cervix Cancer |  |
|  | Cancer, Cervix |  |
|  | Ca cervix |  |
|  | cancer of the cervix uteri |  |
|  | cancer, uterine cervix |  |
|  | carcinogenesis of the cervix |  |
|  | cervical cancerogenesis |  |
|  | cervical carcinogenesis |  |
|  | cervical malignancies |  |
|  | cervical malignancy |  |
|  | cervix ca |  |
|  | cervix cancer, recurrent |  |
|  | cervix cancer, uterine |  |
|  | cervix cancerogenesis |  |
|  | cervix carcinogenesis |  |
|  | cervix malignancies |  |
|  | cervix malignancy |  |
|  | cervix malignancy, recurrent |  |
|  | cervix uteri cancer |  |
|  | cervix uterus cancer |  |
|  | malignancies of the cervix |  |
|  | malignancy of the cervix |  |
|  | neoplasma cervicis recurrens |  |
|  | neoplasma cervicis uteri recurrens |  |
|  | recurrent cancer of the cervix |  |
|  | recurrent cervix cancer |  |
|  | recurrent cervix malignancy |  |
|  | uterine cervix cancer, recurrent |  |
|  | uterine cervix malignancy, recurrent |  |
|  | uterine neck cancer |  |
|  | uterus cervix cancer |  |
|  | uterine cervix cancer |  |

**Supplementary Table S2** Assessment of the risk of bias in each cohort study using the Newcastle–Ottawa scale.

| **Study ID** | **Country** | **Selection** | | | | **Comparability** | | **Exposure** | | | **Total** |
| --- | --- | --- | --- | --- | --- | --- | --- | --- | --- | --- | --- |
|  |  | **1.1** | **1.2** | **1.3** | **1.4** | **2.1** | **2.2** | **3.1** | **3.2** | **3.3** |  |
| Cao 2020 | China | 1 | 1 | 1 | 1 | 0 | 0 | 1 | 1 | 1 | 7 |
| Carus 2014 | Denmark | 1 | 1 | 1 | 1 | 1 | 0 | 1 | 1 | 1 | 8 |
| Chen 2017 | China | 1 | 1 | 1 | 1 | 0 | 0 | 1 | 1 | 1 | 7 |
| Chen 2019 (1) | China | 1 | 1 | 1 | 1 | 0 | 0 | 1 | 1 | 1 | 7 |
| Chen 2019 (2) | China | 1 | 1 | 1 | 1 | 0 | 0 | 1 | 1 | 1 | 7 |
| Davidson 1999 | Israel | 1 | 1 | 1 | 1 | 0 | 0 | 1 | 1 | 0 | 6 |
| Ding 2014 (1) | China | 1 | 1 | 1 | 1 | 0 | 0 | 1 | 1 | 1 | 7 |
| Ding 2014 (2) | China | 1 | 1 | 1 | 1 | 0 | 0 | 1 | 1 | 1 | 7 |
| Ding 2020 | China | 1 | 1 | 1 | 1 | 1 | 0 | 1 | 1 | 1 | 8 |
| Guo 2020 | China | 1 | 1 | 1 | 1 | 1 | 0 | 1 | 1 | 0 | 7 |
| Guo 2021 (1) | China | 1 | 1 | 1 | 1 | 0 | 0 | 1 | 1 | 1 | 7 |
| Kawachi 2018 | Japan | 1 | 1 | 1 | 1 | 0 | 0 | 1 | 1 | 0 | 6 |
| Li 2014 | China | 1 | 1 | 1 | 1 | 0 | 0 | 1 | 1 | 1 | 7 |
| Li 2017 (1) | China | 1 | 1 | 1 | 1 | 1 | 0 | 1 | 1 | 1 | 8 |
| Li 2017 (2) | China | 1 | 1 | 1 | 1 | 0 | 0 | 1 | 1 | 1 | 7 |
| Liu 2005 | China | 1 | 1 | 1 | 1 | 0 | 0 | 1 | 1 | 1 | 7 |
| Liu 2018 | China | 1 | 1 | 1 | 1 | 0 | 0 | 1 | 1 | 1 | 7 |
| Liu 2020 | China | 1 | 1 | 1 | 1 | 0 | 0 | 1 | 1 | 1 | 7 |
| Liu 2021 | China | 1 | 1 | 1 | 1 | 0 | 0 | 1 | 1 | 1 | 7 |
| Ohno 2020 | Japan | 1 | 1 | 1 | 1 | 0 | 0 | 1 | 1 | 1 | 7 |
| Wang 2018 | China | 1 | 1 | 1 | 1 | 1 | 0 | 1 | 1 | 1 | 8 |
| Wang 2023 | China | 1 | 1 | 1 | 1 | 1 | 0 | 1 | 1 | 0 | 7 |
| Yan 2018 | China | 1 | 1 | 1 | 1 | 0 | 0 | 1 | 1 | 1 | 7 |
| Zheng 2013 | China | 1 | 1 | 1 | 1 | 0 | 0 | 1 | 1 | 1 | 7 |
| Zhou 2018 | China | 1 | 1 | 1 | 1 | 1 | 0 | 1 | 1 | 0 | 7 |
| Zou 2021 | China | 1 | 1 | 1 | 1 | 1 | 0 | 1 | 1 | 0 | 7 |

1.1Adequate definition of cases; 1.2Representativeness of the cases; 1.3Selection of Controls; 1.4Definition of Controls; 2.1Comparability of cases and controls on the basis of the design or analysis 1; 2.2Comparability of cases and controls on the basis of the design or analysis 2; 3.1Ascertainment of exposure; 3.2Same method of ascertainment for cases and controls; 3.3Non-Response rate.

**Supplementary Table S3** Results of subgroup analysis, sensitivity analysis and publication bias test

| **Analysis** | **Number** | **Participants** | **Random-effects model** | | **Fixed-effects model** | | **Heterogeneity** | | **Egger's test** |
| --- | --- | --- | --- | --- | --- | --- | --- | --- | --- |
|  |  |  | **Effect size [95%CI]** | ***P*** | **Effect size [95%CI]** | ***P*** | **I^2^** | ***P*** | ***P*** |
| **Expression in CC/N** | **7** | **551** | **12.12 [7.14, 20.58]** | **0** | **12.93 [7.73, 21.61]** | **0** | **2** | **0.41** |  |
| CD68 | 5 | 423 | 9.96 [5.49, 18.05] | 0 | 10.90 [6.04, 19.64] | 0 | 45 | 0.18 |  |
| CD163 | 2 | 128 | 28.12 [5.38, 147.04] | 0 | 23.97 [8.42, 68.26] | 0 | 0 | 0.62 |  |
| **Density in CC or N** | **12** | **1042** | **1.58 [0.95, 2.21]** | **0** | **1.35 [1.20, 1.50]** | **0** | **94** | **0** | **0.272** |
| CD68 | 7 | 522 | 1.56 [0.39, 2.73] | 0 | 1.12 [0.90, 1.34] | 0 | 96 | 0 |  |
| CD163 | 5 | 520 | 1.59 [1.34, 1.84] | 0.009 | 1.57 [1.35, 1.78] | 0 | 21 | 0.28 |  |
| **LNM** | **25** | **2544** | **0.51 [0.32, 0.71]** | **0** | **0.44 [0.35, 0.54]** | **0** | **74** | **0** | **0.106** |
| CD68 | 15 | 1476 | 0.44 [0.20, 0.68] | 0.0004 | 0.42 [0.30, 0.55] | 0 | 71 | 0 |  |
| CD163 | 9 | 1023 | 0.49 [0.20, 0.79] | 0.001 | 0.43 [0.27, 0.58] | 0 | 68 | 0.002 |  |
| CD206 | 1 | 45 | 2.56 [1.58, 3.53] | 0 | 2.56 [1.58, 3.53] | 0 |  |  |  |
| TS | 14 | 1020 | 0.58 [0.31, 0.85] | 0 | 0.44 [0.30, 0.59] | 0 | 70 | 0 |  |
| TC | 6 | 1120 | 0.19 [-0.11, 0.50] | 0.22 | 0.36 [0.20, 0.51] | 0 | 70 | 0.006 |  |
| S 1-2 | 8 | 413 | 1.21 [0.75, 1.66] | 0 | 1.08 [0.86, 1.31] | 0 | 74 | 0.0003 |  |
| S 1-4 | 15 | 2011 | 0.24 [0.08, 0.39] | 0.003 | 0.29 [0.18, 0.40] | 0 | 45 | 0.03 |  |
| **FIGO** | **14** | **786** | **0.46 [0.08, 0.85]** | **0.02** | **0.38 [0.22, 0.54]** | **0** | **82** | **0** | **0.267** |
| CD68 | 9 | 517 | 0.15 [-0.22, 0.52] | 0.43 | 0.16 [-0.04, 0.37] | 0.11 | 68 | 0.002 |  |
| CD163 | 4 | 224 | 0.71 [0.10, 1.32] | 0.02 | 0.51 [0.24, 0.79] | 0.0002 | 78 | 0.004 |  |
| CD206 | 1 | 45 | 2.49 [1.70, 3.29] | 0 | 2.49 [1.70, 3.29] | 0 |  |  |  |
| **Age** | **13** | **770** | **-0.01 [-0.16, 0.13]** | **0.84** | **-0.01 [-0.16, 0.13]** | **0.84** | **0** | **0.7** | **0.913** |
| CD68 | 8 | 487 | -0.06 [-0.24, 0.12] | 0.51 | -0.06 [-0.24, 0.12] | 0.51 | 0 | 0.45 |  |
| CD163 | 5 | 283 | 0.07 [-0.17, 0.31] | 0.58 | 0.07 [-0.17, 0.31] | 0.58 | 0 | 0.82 |  |
| **HPV** | **5** | **561** | **0.02 [-0.24, 0.28]** | **0.86** | **0.02 [-0.24, 0.28]** | **0.86** | **0** | **1** |  |
| CD68 | 3 | 346 | 0.01 [-0.30, 0.33] | 0.93 | 0.01 [-0.30, 0.33] | 0.93 | 0 | 0.98 |  |
| CD163 | 2 | 215 | 0.04 [-0.41, 0.49] | 0.86 | 0.04 [-0.41, 0.49] | 0.86 | 0 | 0.92 |  |
| **Tumor size** | **7** | **584** | **0.31 [-0.18, 0.81]** | **0.21** | **0.09 [-0.08, 0.26]** | **0.29** | **87** | **0** |  |
| CD68 | 3 | 269 | -0.02 [-0.27, 0.22] | 0.84 | -0.02 [-0.27, 0.22] | 0.84 | 0 | 0.76 |  |
| CD163 | 2 | 100 | 0.00 [-0.27, 0.27] | 0.98 | 0.00 [-0.27, 0.27] | 0.98 | 0 | 0.98 |  |
| CD206 | 1 | 100 | 1.58 [-1.71, 4.88] | 0.35 | 0.87 [0.37, 1.36] | 0 | 97 | 0 |  |
| **OS** | **9** |  | **2.55 [1.59, 4.07]** | **0** | **2.63 [2.11, 3.29]** | **0** | **71** | **0.0005** |  |
| CD68 | 4 |  | 2.58 [1.40, 4.76] | 0.002 | 2.72 [1.98, 3.75] | 0 | 66 | 0.03 |  |
| CD163 | 3 |  | 3.55 [1.47, 8.58] | 0.005 | 2.77 [2.00, 3.82] | 0 | 81 | 0.0005 |  |
| CD204 | 1 |  | 1.88 [0.61, 5.79] | 0.27 | 1.88 [0.61, 5.79] | 0.27 |  |  |  |
| CD206 | 1 |  | 0.06 [0.00, 0.84] | 0.04 | 0.06 [0.00, 0.84] | 0.04 |  |  |  |
| **RFS** | **3** |  | **2.17 [1.40, 3.35]** | **0.0005** | **2.17 [1.40, 3.35]** | **0.0005** | **0** | **0.92** |  |

**
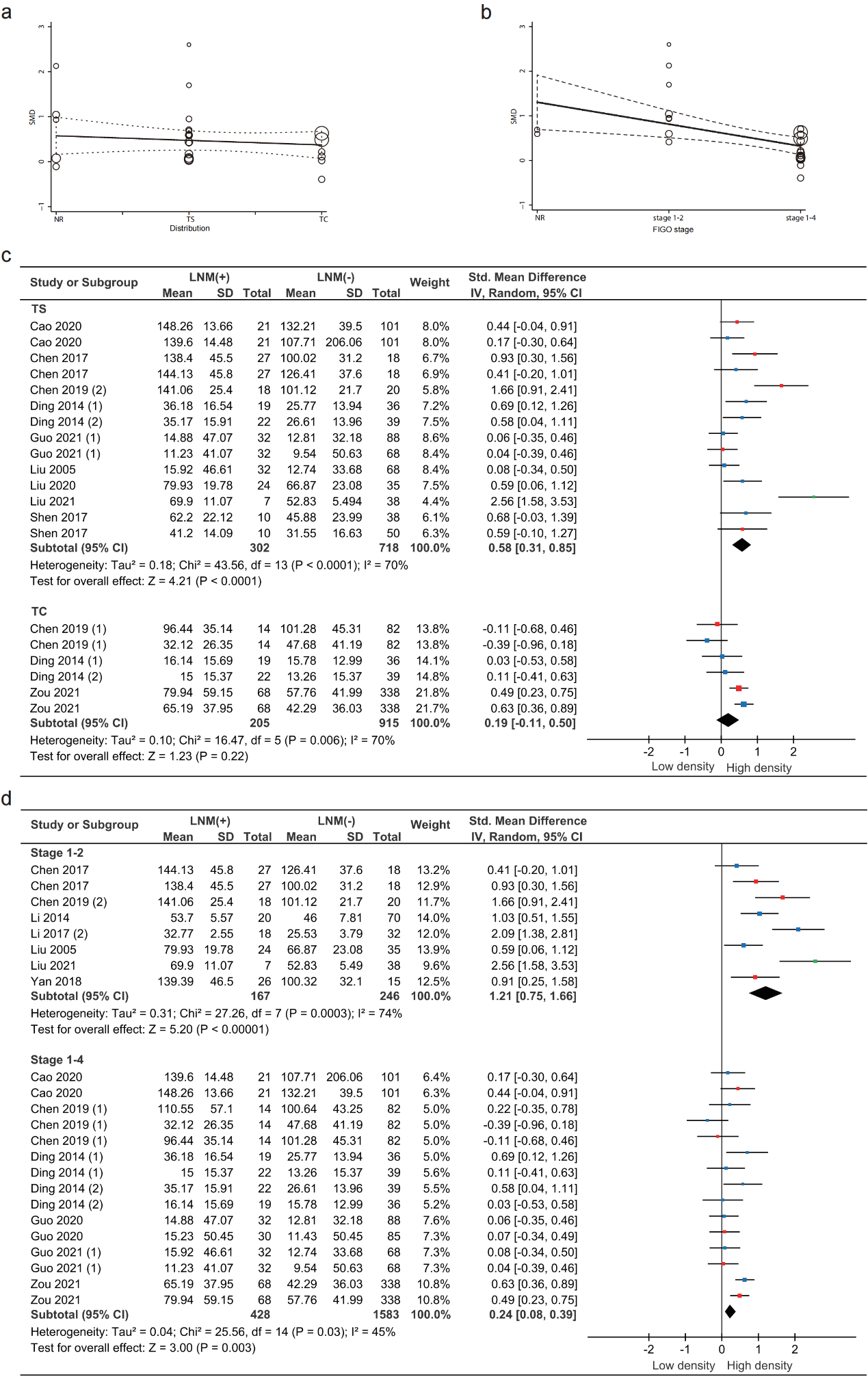
**

**Supplementary Fig. S1** Forest plot of TAMs density and LNM. (a) Meta-regression analysis of TAMs density in TS or TC. (b) Meta-regression analysis of TAMs density in stage1-2 or stage1-4 CC. (c) TAMs density in TS or TC. (d) TAMs density in stage1-2 or stage1-4 CC.

**
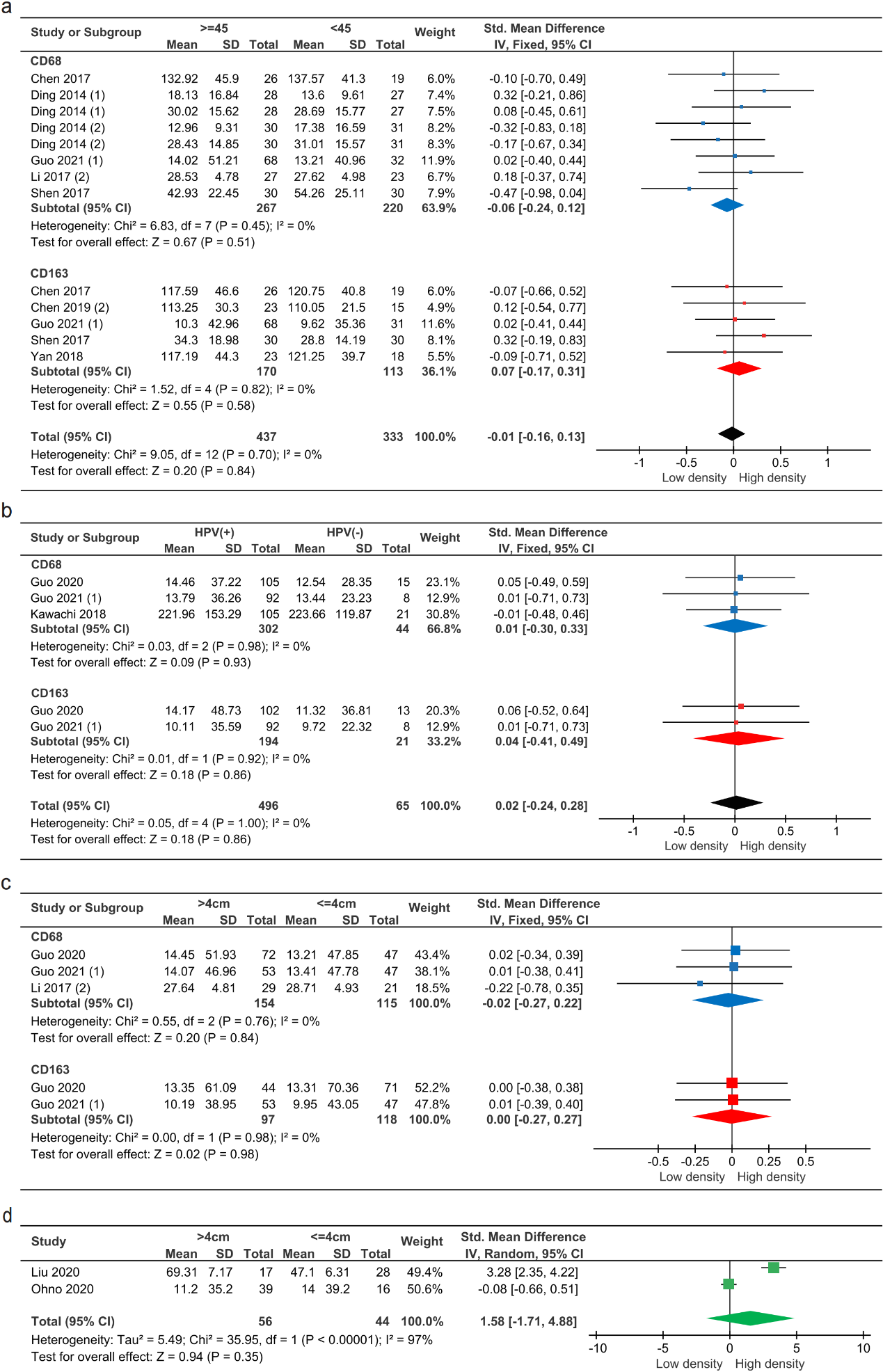
**

**Supplementary Fig. S2** Forest plot of TAMs density and clinicopathological features. (a) TAMs density and age. (b) TAMs density and HPV infection status. (c) (d) TAMs density and tumor size.

**
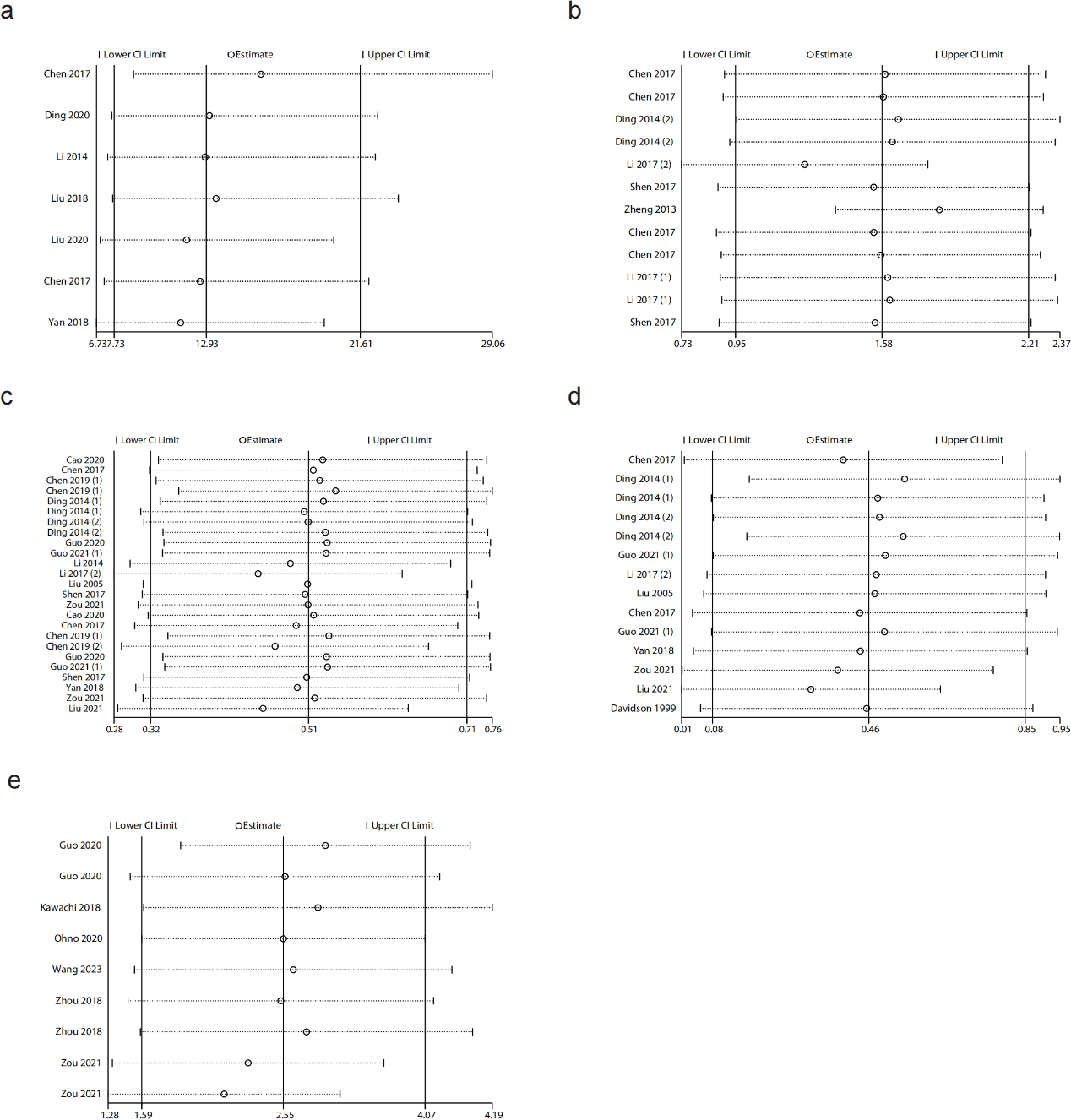
**

**Supplementary Fig. S3** Sensitivity analysis showed that the results of were stable. (a) TAMs expression in CC or normal. (b) TAMs density in CC or normal. (c)TAMs density and LNM. (d) TAMs density and FIGO stage. (E) TAMs density and OS.


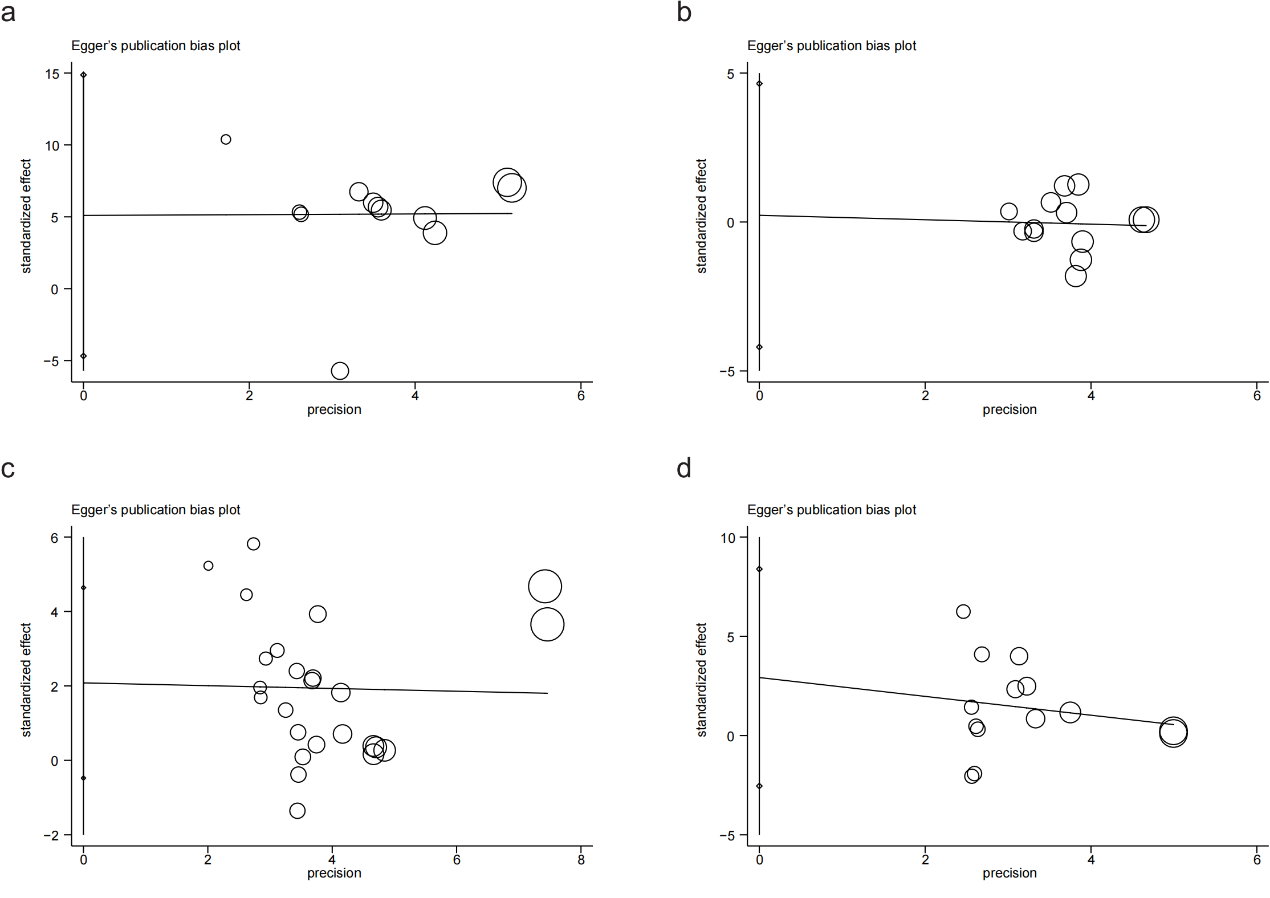


**Supplementary Fig. S4** Egger’s funnel plot showed no publication bias among the included studies. (a) TAMs density in CC or normal (*P* = 0.272). (b) TAMs density and LNM (*P* = 0.106). (c) TAMs density and age (*P* = 0.913). (d) TAMs density and FIGO stage (*P* = 0.267).
